# SARS-CoV-2 Omicron Variants Reduce Antibody Neutralization and Acquire Usage of Mouse ACE2

**DOI:** 10.3389/fimmu.2022.854952

**Published:** 2022-06-17

**Authors:** Ruoke Wang, Qi Zhang, Rui Zhang, Zhen Qin Aw, Peng Chen, Yi Hao Wong, Junxian Hong, Bin Ju, Xuanling Shi, Qiang Ding, Zheng Zhang, Justin Jang Hann Chu, Linqi Zhang

**Affiliations:** ^1^ Center for Global Health and Infectious Diseases, Comprehensive AIDS Research Center, Department of Basic Medical Sciences, School of Medicine, Tsinghua University, Beijing, China; ^2^ Tsinghua-Peking Joint Center for Life Sciences, Beijing, China; ^3^ Biosafety Level 3 Core Facility, Yong Loo Lin School of Medicine, National University of Singapore, Singapore, Singapore; ^4^ Laboratory of Molecular RNA Virology and Antiviral Strategies, Department of Microbiology and Immunology, Yong Loo Lin School of Medicine, National University of Singapore, Singapore, Singapore; ^5^ Infectious Disease Translation Research Programme, Yong Loo Lin School of Medicine, National University of Singapore, Singapore, Singapore; ^6^ Institute for Hepatology, National Clinical Research Center for Infectious Disease, Shenzhen Third People’s Hospital, The Second Affiliated Hospital, School of Medicine, Southern University of Science and Technology, Shenzhen, China; ^7^ The Second Affiliated Hospital, School of Medicine, Southern University of Science and Technology, Shenzhen, China; ^8^ Center for Infectious Disease Research, School of Medicine, Tsinghua University, Beijing, China; ^9^ Beijing Advanced Innovation Center for Structural Biology, Tsinghua University, Beijing, China; ^10^ Institute of Biopharmaceutical and Health Engineering, Tsinghua Shenzhen International Graduate School, Tsinghua University, Shenzhen, China; ^11^ Shenzhen Bay Laboratory, Institute of Biomedical Health Technology and Engineering, Shenzhen, China

**Keywords:** Omicron variant, antibody neutralization, ACE2 orthologues, SARS-CoV-2 infection (COVID-19), plasma neutralization

## Abstract

Striking number of mutations found in the spike protein of recently emerged SARS-CoV-2 Omicron subvariants BA.1, BA.2, BA.3 and BA.4/5 has raised serious concerns regarding the escape from current antibody therapies and vaccine protection. Here, we conducted comprehensive analysis on the extent of two major Omicron lineages BA.1/BA.1.1 and BA.2 to escape neutralization from the therapeutic antibodies approved by the regulatory authorities and convalescent plasma from SARS-CoV-2 patients infected during initial wave of pandemic in early 2020. We showed that Omicron BA.1/BA.1.1 were the most resistant in both magnitude and breadth against antibodies and convalescent plasma, followed by Beta, BA.2, Gamma, Delta and Alpha. While the majority of therapeutic antibodies lost binding and neutralization to Omicron variants, BRII combo (BRII-196 + BRII-198), S309, and AZ combo (COV2-2196 + COV2-2130) maintained neutralization despite of reduction due to either conserved epitope or combinational effect between the two designated antibodies. A single intraperitoneal injection of BRII combo as a prophylactic treatment protected animals from Omicron infection. Treated animals manifested normal body weight, survived infection up to 14 days, undetectable levels of infectious viruses in the lungs, and reduced lung pathology compared to the controls. Analyzing ACE2 from diverse host species showed that Omicron variants acquired ability to use mouse ACE2 for entry. These results demonstrate major antigenic shifts and potentially broadening the host range of two major Omicron lineages BA.1/BA.1.1 and BA.2, posing serious challenges to current antibody therapies and vaccine protection as well as increasing danger of spillover into the wildlife.

## Introduction

As the severe acute respiratory syndrome coronavirus 2 (SARS-CoV-2) continues to rage around the world, we have witnessed the rapid emergence and turnover of multiple variants of concerns (VOCs) such as Alpha (B.1.1.7), Beta (B.1.351), Gamma (P.1), Delta (B.1.617.2), and Omicron (BA.1/BA.1.1 BA.2, BA.3, and BA.4/5). There is growing concern that they could become antigenically distinct from the original strain to the extent that render current therapeutic antibody and vaccine strategies ineffective. Among the previous identified Alpha, Beta, Gamma, and Delta VOCs, Beta is the most discrete in antigenic properties which leads to the substantial reduction in its sensitivity to therapeutic antibodies and plasma neutralization from convalescent and vaccinated individuals (1–5). However, Beta appears to be less transmissible and has mostly been circulating in South Africa since it was initially identified there. By contrast, Delta has spread far beyond original country of India and continues dominating in many parts of the world due to its superior transmissibility albeit relative minor changes in antigenicity (6–11).

Recently, increased attention has been paid to Omicron variants (BA.1, BA.2, BA.3 and BA.4/5), the new member of VOCs that was initially identified in November 2021 in Botswana and South Africa (WHO or CDC) (12–14). According to the recent report from the World Health Organization (WHO) (8), Omicron variants have already spread to many countries around world and is associated with steeply increased infections among unvaccinated population as well as breakthrough infections among vaccinated individuals (15–17). One alarming aspect of Omicron variants is the largest number of mutations found in the S protein among all VOCs identified so far (**Supplementary Figure 1**). It is currently unknow how Omicron accumulated such high number of mutations in such a short period, although some speculated that it may derive from immunocompromised individuals or spillback from other animal species (18, 19). A total of 32 mutations in the spike (S) protein has been found in the predominant Omicron strain. Of which, at least 15 substitutions are located in the receptor-binding domain (RBD) and 8 substitutions and insertons/deletions are in the N-terminal domain (NTD) (**Supplementary Figure 1**). As RBD and NTD are the major target of neutralizing antibodies upon infection and vaccination, these changes may result in Omicron’s escape from antibody treatment and vaccine protection. Indeed, preliminary results indicate that two major lineages of Omicron (BA.1/BA.1.1 and BA.2) can escape from some of the approved therapeutic antibodies under emergency use authorization (EUA) (18, 20–27). Serum neutralizing activities from convalescent and vaccinated individuals are also severely compromised regardless of infection status or type of vaccines used (17, 18, 20–23, 25, 28–32). However, to what extent Omicron lineages BA.1/BA.1.1, and BA.2 could escape therapeutic antibodies and convalescent plasma from the early wave of SARS-CoV-2 pandemic remains unclear. The impact of highly mutated BA.1/BA.1.1 and BA.2 spike on its interaction with ACEs from human and diverse animal hosts is also unclear. Here, we show that two major lineages of Omicron BA.1/BA.1.1 and BA.2 substantially reduces neutralization of majority of therapeutic antibodies and convalescent plasma collected during the early pandemic. However, despite of reduction, BRII combo (BRII-196 + BRII-198), S309, and AZ combo (COV2-2196 + COV2-2130) maintained neutralizing activity, perhaps due to conserved epitope or combinational effect between the two designated antibodies. Furthermore, a single intraperitoneal injection of BRII combo protected animals from Omicron infection, suggesting its neutralizing activity *in vitro* could be translated into protectivity *in vivo* in this animal model. Finally, Omicron lineages BA.1/BA.1.1 and BA.2 also acquire ability to use mouse ACE2 for entry. These results clearly show major antigenic shifts and potentially expanding the host range of Omicron BA.1/BA.1.1 and BA.2, posing serious challenges to antibody and vaccine protection as well as further spread into the wildlife.

## Results

### Substantial Reduction in Antibody Neutralization of Omicron BA.1, BA.1.1 and BA.2

**Figure 1:**
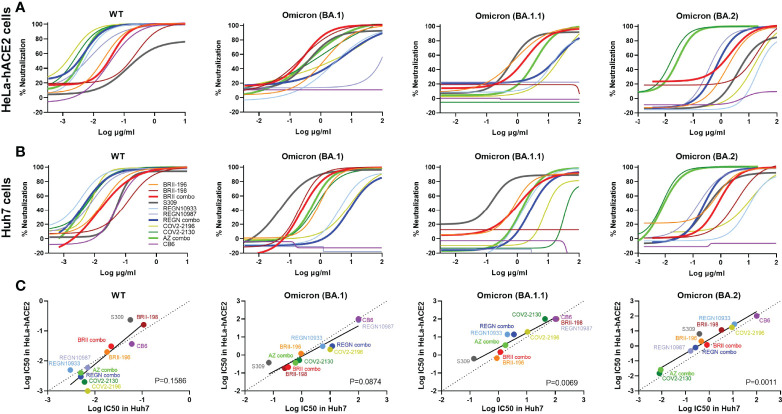
Substantial reduction in antibody neutralization to Omicron BA.1, BA.1.1, and BA.2. Neutralizing activity of each therapeutic antibody and their designated combinations to wildtype (WT), Omicron BA.1, BA.1.1, and BA.2 pseudoviruses analyzed in **(A)** HeLa cells expressing human ACE2 (HeLa-hACE2) and **(B)** in Huh7 cells. WT and Omicron pseudoviruses were tested against serial dilutions of each antibody and relevant combinations. Neutralizing activity was defined as the percent reduction in luciferase activities compared to no antibody controls. Results were derived from two independent experiments and each included two technical replicates. **(C)** Correlation between Log IC50 for all tested mAbs and their clinical-relevant combinations in HeLa-hACE2 and Huh7 cells. The R^2^ and P values of correlation were 0.7963 and 2.2e-4 for WT, 0.8768 and 2.2e-5 for BA.1, 0.9017 and 7.9e-6 for BA.1.1, and 0.9374 and 1.0e-6 for BA.2, determined by two-tailed Spearman correlation. Linear regression of experimental Log IC50 was estimated (solid line) and compared with a hypothetical regression (dotted line) for assumption of equal IC50s in both HeLa-hACE2 and Huh7 cell lines. No significant differences were detected between experimental and hypothetical regression for WT (P = 0.1586) and BA.1 (P = 0.0874), but significantly lower levels in Huh7 cells was found than in HeLa-hACE2 cells for BA.1.1 (P = 0.0069) and BA.2 (P = 0.0011).

To study the impact of Omicron subvariants BA.1, BA.1.1, and BA.2 on antibody neutralization, we focused on antibodies approved by the regulatory agencies such as BRII-196 (also known as amubarvimab) and BRII-198 (also known as romlusevimab) developed by Brii Biosciences, S309 (the parental antibody of sotrovimab) by GlaxoSmithKline and Vir Biotechnology, REGN10933 (also known as casirivimab) and REGN10987 (also known as imdevimab) by Regeneron, COV2-2196 (the parental antibody of AZD8895) and COV2-2130 (the parental antibody of AZD1061) by AstraZeneca, and CB6 (the parental antibody of etesevimab) by Eli Lilly. We chose to test these antibodies first against Omicron subvariants BA.1, BA.1.1, and BA.2 and compared with that against original wildtype strain Wuhan-Hu-1 (WT, Genbank reference MN908947) as they had well been studied already against previously identified VOCs such as Alpha, Beta, Gamma and Delta (1, 2, 33–37). Pseudoviruses bearing the spike protein of Omicron variants BA.1, BA.1.1, BA.2, and WT were subjected to neutralization analysis against 8 therapeutic antibodies individually and in combination as developed clinically (**Figure 1** and **Table 1**). To improve assay representation, the neutralization was conducted in two different cell lines: HeLa cells stably expressing human ACE2 (HeLa-hACE2) and Huh7 cells previously used for SARS-CoV-2 infection and neutralization (1, 38). In HeLa-hACE2 cell line, BRII combo (BRII-196 + BRII-198), S309, and AZ combo (COV2-2196 and COV2-2130) maintained neutralizing activity below single-digit μg/mL concentration. The rest antibodies, however, demonstrated substantially reduced or lost activity against all three Omicron variants (**Figure 1A** and **Table 1**). Among the three Omicron variants, BA.1.1 had the most adverse effect on BRII combo and AZ combo, resulting in approximately 47.4- and 912.5-fold reduction in IC50, relative to the WT (**Table 1**). BA.2, on the other hand, was the most disruptive against S309 and reduced its IC50 by about 27.4-fold (**Table 1**). Interestingly, despite of marked reduction in neutralization for individual COV2-2196 and COV2-2130, AZ combo regained neutralization to Omicron with IC50 of 0.370 μg/mL to BA.1, 3.493 μg/mL to BA.1.1, and 0.026 μg/mL to BA.2, perhaps due to the synergistic effect between the two antibodies (**Figure 1A** and **Table 1**). In Huh7 cells, the estimated IC50 and fold changes relative to WT remained largely consistent with that in HeLa-hACE2 cells (**Figure 1B** and **Table 1**). BRII combo, S309, and AZ combo maintained similar trend against the Omicron strains tested, although all of them appeared to perform better in Huh7 cells than in HeLa-hACE2 cells. This was particular true for S309 where its respective IC50 to BA.1, BA.1.1 and BA.2 improved from 0.376 μg/mL, 0.629 μg/mL, and 6.438 μg/mL in HeLa-hACE2 cells to 0.069 μg/mL (5.4-fold), 0.137 μg/mL (4.6-fold), and 0.378 μg/mL (17.0-fold) in Huh7 cells. This is consistent with previous finding that neutralizing activity of S309 varied in cell lines overexpressing ACE2 (39). For BRII combo, BRII-196, and BRII-198, neutralizing activity against live authentic Omicron BA.1 virus demonstrated the similar trend with IC50 of 0.168 μg/mL, 2.370 μg/mL and 0.632 μg/mL, respectively (**Supplementary Figure 2**). Furthermore, when comparing the linear regression between experimental IC50 values of all tested mAbs and their clinical-relevant combinations (solid line) and hypothetical regression (dotted line) for assumption of equal IC50s in both cell lines, we found no significant differences for WT (P=0.1586) and BA.1 (P=0.0874), but significantly higher activity in Huh7 than in HeLa-hACE2 cells for BA.1.1 (P=0.0069) and BA.2 (P=0.0011) (**Figure 1C**). These results indicate that substantial differences do exist between different cell lines which need to be taken into account when interpreting the antibody neutralizing activity.

**Table 1:** Neutralizing and binding activities of therapeutic antibodies against WT and Omicron variants.

	Neut in Hela-hACE2 cells							Neut in Huh 7 cells							Fold Change of Spike binding		
	IC50 (µg/ml)				Fold Change			IC50 (µg/ml)				Fold Change					
	WT	BA.1	BA.1.1	BA. 2	BA .1	BA. 1. 1	BA. 2	WT	BA.1	BA.1.1	BA. 2	BA .1	BA. 1. 1	BA. 2	BA .1	BA. 1. 1	BA. 2
BRII-196	0.020	1.210	0.665	2.087	-61.5	-33.8	-106.1	0.018	0.949	0.877	0.463	-52.5	-48.5	-25.6	-2.0	-2.1	-4.3
BRII-198	0.162	0.184	>100	11.690	-1.1	<-617.6	-72.2	0.107	0.251	>100	3.303	-2.4	<-938.6	-31.0	+4.2	-198.9	-19.8
BRII-Combo	0.031	0.211	1.467	1.177	-6.8	-47.4	-38.1	0.022	0.332	1.168	0.801	-15.5	-54.3	-37.3	+1.9	-2.2	-3.9
S309	0.235	0.376	0.629	6.438	-1.6	-2.7	-27.4	0.056	0.069	0.137	0.387	-1.2	-2.4	-6.9	-1.3	-1.2	-3.4
REGN 10933	0.005	3.058	13.939	28.239	-625.9	-2852.7	-5779.1	0.003	5.463	2.055	11.507	-1959.2	-737.1	-4126.6	-5.8	-4.4	-7.3
REGN 10987	0.006	85.540	>100	0.465	-14398.4	<-16832.3	-78.3	0.007	>100	>100	0.163	<-13402.2	<-13402.2	-21.9	-70.7	-34.7	-1.4
REGN Combo	0.003	3.131	13.890	0.794	-962.9	-4271.2	-244.2	0.005	11.958	3.523	0.264	-2468.0	-727.1	-54.4	-7.0	-6.8	-1.3
COV2-2196	0.001	2.048	19.246	17.467	-1517.5	-1426.6	-12942.9	0.007	9.977	10.494	9.089	1387.9	-1459.8	-1264.3	+1.5	+1.6	-1.3
COV2-2130	0.002	0.512	>100	0.015	-233.0	<-45499.2	-6.9	0.006	0.818	43.402	0.008	-140.9	-7478.3	-1.4	-1.3	-6.0	-1.0
AZ como	0.004	0.370	3.493	0.026	-96.7	-912.5	-6.7	0.005	0.600	1.755	0.009	-113.4	-331.6	-1.7	+1.2	+1.1	+1.0
CB6	0.037	>100	>100	>100	<-2734.9	<-2734.9	<-2734.9	0.060	>100	>100	>100	<-1662.2	<-1662.2	<-1662.2	<-200	<-200	<-200

### Substantial Reduction in Antibody Binding to Omicron BA.1, BA.1.1, and BA.2

**Figure 2:**
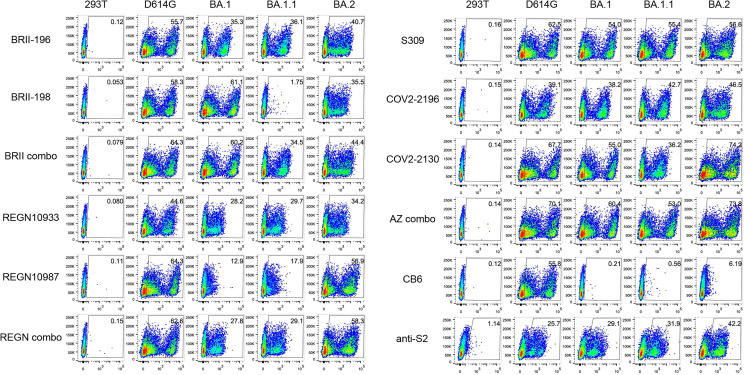
Antibody binding to spikes of Omicron BA.1, BA.1.1, and BA.2 expressed on the cell surface. Binding of all tested mAbs, and their clinical-relevant combinations to the spike protein of WT D614G, Omicron BA.1, BA.1.1, and BA.2 expressed on the surface of HEK293T, measured by flow cytometry. Anti-S2 is a S2-specific antibody used for positive control. The numbers highlighted in the gates represent the percent of positive cells detected by indicated antibodies or combinations. The result shown was representative of two independent experiments.

We next studied the binding activity of these antibodies individually or in combination to the spike protein of the three Omicron variants expressed on the surface of HEK293T (**Figure 2**). The fold-changes in normalized total fluorescence intensity (nTFI) relative to that of D614G spike were calculated and indicated in **Table 1**. Consistent with neutralization activity, BRII combo, S309, and AZ combo maintained binding to all three variants with less than five-fold reduction, although some of the antibodies, when tested singly, either markedly reduced or lost binding to at least one of the variants tested (**Figure 2**). BRII combo demonstrated somewhat increased binding to BA.1 (+1.9-fold) while decreased binding to BA.1.1 (-2.2-fold) and BA.2 (-3.9-fold), relative to WT (**Table 1**). S309 reduced binding to the spike protein of all three variants, most notably to BA.2 (-3.4-fold) (**Table 1**). By contrast, AZ combo demonstrated relatively consistent binding to BA.1 (+1.2-fold), BA.1.1 (+1.1-fold), and BA.2 (+1.0-fold), providing some mechanistic basis for its synergistic neutralizing activity, particularly against BA.2 (**Table 1** and **Figure 1**). This was perhaps owing to specific mutations in BA.2 restored the neutralizing activity of COV2-2130 (**Figure 2** and **Table 1**). These results suggest a relation between antibody binding and neutralization and reduced or lost neutralization to the three Omicron variant was largely attributed to the reduced binding to the spike protein.

### BRII Combo Protects K18-hACE2 Mice From Infection With Authentic SARS-CoV-2 Omicron

**Figure 3:**
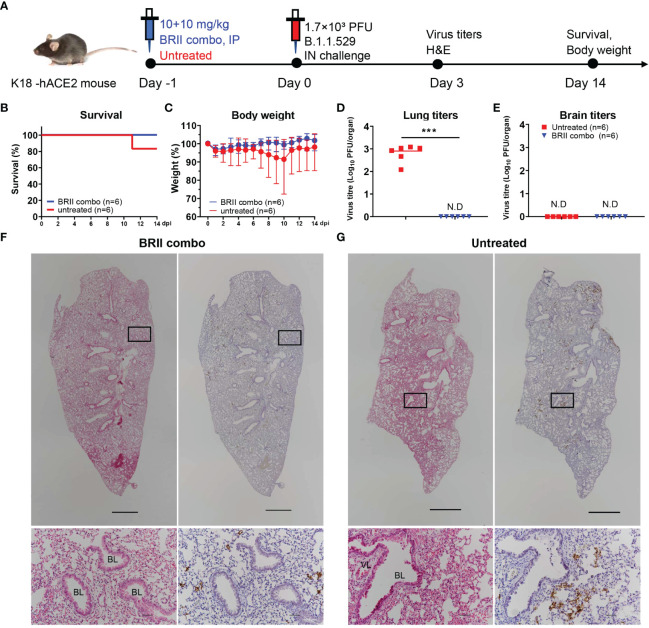
BRII combo protects K18-hACE-2 mice from the infection of authentic SARS-CoV-2 Omicron. **(A)** Experimental schedule for BRII combo prophylaxis. Eight-week-old K18-hACE2 transgenic female mice were administered with 20 (10 + 10) mg/kg body weight of BRII combo (BRII-196 + BRII-198) intraperitoneally or remained untreated. One day later, all animals were challenged with 1.7×10^3^ plaque-forming units (PFU) infectious SARS-CoV-2 Omicron *via* the intranasal route. **(B)** The survival percentage and **(C)** body-weight were recorded daily after infection until the occurrence of death or until the end of experiment. The viral load in **(D)** the lung and **(E)** the brain tissues was measured by plaque forming assays in the tissue homogenates at 3 days post inoculation. Data are presented as the means ± SEM. N.D: not detected. Mann-Whitney test was used to analyze statistical significance. ***P < 0.001. **(F, G)** H&E and immunohistochemistry staining of lung tissue from BRII combo-treated or untreated mice at 3 days post inoculation. The upper panels show the whole lung sections (5x; Scale bars=1000µm) while the lower panels displayed the enlarged view of the boxed regions (50x; Scale bars=50µm). Dark brown in the enlarged view are SARS-CoV-2 N protein positive cells. VL, vascular lumen; BL, bronchiolar lumen. Images were derived from one representative animal in each group.

We next studied the protective potential of BRII combo against infection of authentic Omicron in a K18-hACE2 mouse model of SARS-CoV-2 infection, as previously described (40) (**Figure 3A**). A total of 24 mice were used in the experiment. Of which, 12 mice were intraperitoneally administered with BRII combo at a dose of 10 + 10 mg/kg body weight (BRII-196 + BRII-198) and the other 12 remained untreated. Twenty-four hours later, all animals were intranasally challenged with 1.7×10^3^ plaque-forming units (PFU) of authentic SARS-CoV-2 Omicron and monitored daily throughout the following 14 days for their body weight and survival. Six each from BRII combo treated and untreated animals were euthanized on day 3 post challenge to obtain lung and brain tissues for viral load and histopathological analysis. As shown in **Figure 3B**, BRII combo treated animals remained healthy and survived infection while one of the six untreated animals succumbed to disease on day 11 after challenge. The body weight followed the similar changes, with moderate increase in BRII combo treated animals while minor loss in untreated animals (**Figure 3C**). In BRII combo treated animals, no detectable levels of live viruses were found in the lungs on day 3 post challenge. In untreated animals, however, the live virus titer reached an average as high as 10^3^ PFU/tissue (**Figure 3D**). No detectable levels of live viruses were found in the brain in either BRII combo treated nor untreated animals (**Figure 3E**). Immunohistochemistry analysis showed that the lung tissue of BRII combo-treated mice remained intact and scattered virial antigen positive cells could be detected (**Figure 3F**). The lung sections of untreated mice, however, presented moderate damage and inflammation with marked infiltration of inflammatory cells. Infected cells were readily detectable using anti-N protein specific antibody (**Figure 3G**).

### Substantial Reduction in Neutralizing Activity of Convalescent Plasma to Omicron BA.1, BA.1.1 and BA.2

**Figure 4:**
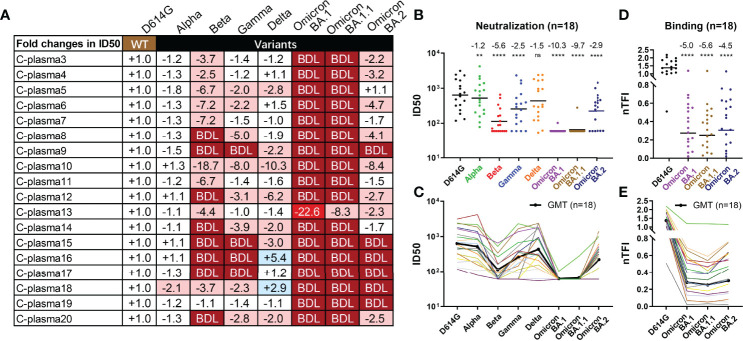
Substantial reduction in neutralizing activity of convalescent plasma to Omicron variants. Reciprocal plasma dilutions (ID50) against SARS-CoV-2 variants are shown either by **(A)** fold changes relative to D614G pseudoviruses, or absolute values in **(B)** colored dots and **(C)** colored curves. The average fold changes in ID50 between each variant and D614G pseudoviruses are shown individually in **(A)** or as a group at the top in **(B)**. The symbol “-” indicates an increase in resistance while the symbol “+” indicates an increase in sensitivity. Those in light red indicate a minimum of 2-fold increase in resistance; dark red a minimum of 20-fold increase in resistance; blue a minimum 2-fold increase in sensitivity; and white a less than 2-fold change in either resistance or sensitivity. BDL (Below Detection Limit) indicates the highest concentration of plasma (1:60 for D614G, Alpha, Beta, and Gamma and 1:20 for Delta and Omicron) failed to confer 50% neutralization. **(B, C)** Each dot or curve represents a different plasma sample. The geometric mean titer of ID50 against each variant is indicated by a black solid line. **(D, E)** Plasma binding to spike proteins on the cell surface measured by FACS and presented as normalized total fluorescent intensity (nTFI) in **(D)** colored dots and **(E)** colored curves. The results were calculated from two independent experiments, and each included two experimental replicates. **P < 0.01; and ****P < 0.0001. ns, not significant.

We next studied to what extent Omicron BA.1, BA.1.1, and BA.2 could escape from neutralization of convalescent plasma collected during the early wave of the pandemic. A total of 18 convalescent plasma samples were obtained between one or two months after wildtype strain Wuhan-Hu-1 infection. Of which, eleven patients had only mild symptoms while the remaining seven developed severe disease. The average age was 54 ranging between 29 and 81 years old. Ten were men and eight were women. For each plasma sample, eight 3-fold serial dilutions were made starting from 1:60 and neutralization activity was estimated based on half-maximal inhibitory dilution (ID50) and fold changes relative to that against D614G pseudovirus (**Figure 4** and **Supplementary Figure 3**). The data on Alpha, Beta, and Gamma have been previously reported (1) and included here for comparison only. A complete loss in neutralization activity (below the limit of detection, BDL) was found in 17 of the 18 plasma samples tested against Omicron BA.1 and BA.1.1, 8 against Beta, 6 against BA.2, 4 against Gamma, and none against Alpha and Delta (**Figure 4A**). The remaining plasma demonstrated varying degree of reduction or increase in neutralization potency against VOCs tested (**Figure 4A**, and **Supplementary Figure 3**). As a result, the greatest reduction in plasma neutralization was against Omicron BA.1 (10.3-fold), followed by BA.1.1 (9.7-fold), Beta (5.6-fold), BA.2 (2.9-fold), Gamma (2.5-fold), Delta (1.5-fold), and Alpha (1.2-fold) (**Figures 4B, C**). Furthermore, we also measured plasma binding to the spike protein of the three Omicron variants expressed on the surface of HEK293T. The fold-changes in normalized total fluorescence intensity (nTFI) relative to that of D614G spike were calculated and presented in **Figures 4D, E**. Consistent with neutralization activity, the plasma binding activity also reduced to the three variants regardless of in absolute values or fold-reduction compared to the WT (**Figures 4D, E**, and **Supplementary Figure 4**). These results clearly show that Omicron BA.1 and BA.1.1 variants were the most resistant followed by BA.2 against the convalescent plasma tested. It is possible the such reduction and loss were attributed to the striking number of mutations found in the Omicron spike including 142‐144del in the NTD and G339D, S371L, S373P, S375F, K417N, E484A, Q493R, and N501Y in the RBD that had previously been shown to confer resistance against antibody and serum neutralization (1–3, 41–43), although other mutations might have made additional contribution (**Supplementary Figure 1**). Interestingly, the reduction in neutralization to BA.2 appeared to be smaller compared to BA.1 and BA.1.1, perhaps due to the relative conserved NTD region compared to BA.1 and BA.1.1 (**Supplementary Figure 1**). Finally, individual convalescent plasma samples appear to respond differently to Omicron variants and other VOCs pseudoviruses, perhaps reflecting their different compositions and proportions of neutralizing antibodies in each individual generated during natural infection.

### Omicron Variant Acquires Usage of Mouse ACE2 for Viral Entry

**Figure 5:**
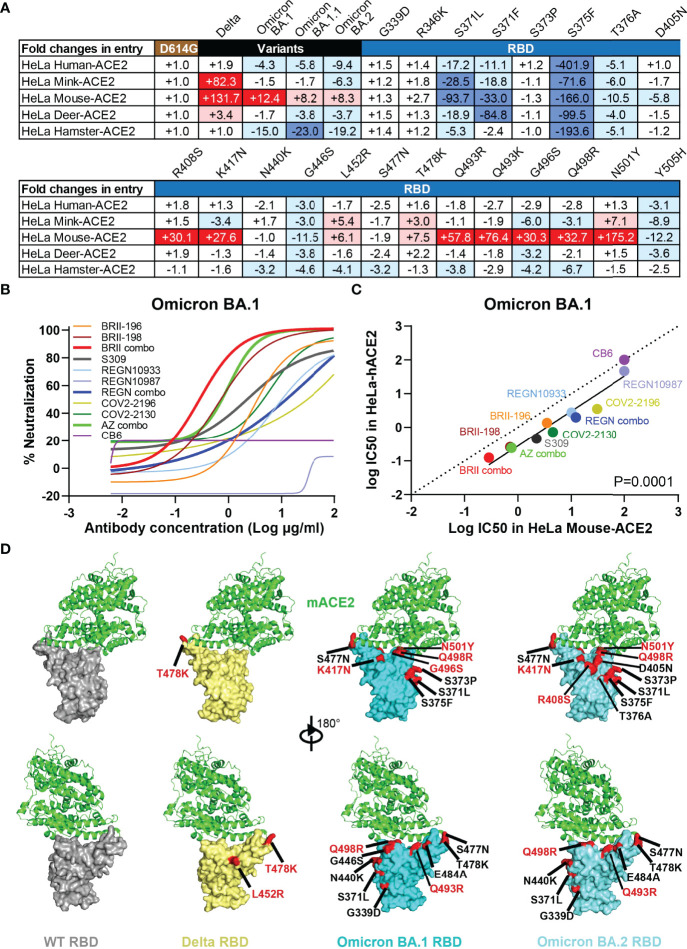
Omicron variants acquire usage of mouse ACE2 and reduces sensitive to antibody neutralization. **(A)** Entry efficiency of SARS-CoV-2 Delta, Omicron BA.1, BA.1.1, BA.2, and 21 single mutant pseudoviruses into HeLa cell lines ectopically expressing various host ACE2. The values represent the fold changes in luciferase activity relative to D614G. The symbol “+” indicates an increase while “-” indicates a decrease in entry efficiency. Red color highlights at least threefold increase in entry efficiency; blue indicates at least threefold decrease in efficiency, while white indicates no change greater than threefold. Results were derived from two independent experiments. **(B)** Neutralizing sensitivity of Omicron BA.1 to each therapeutic antibody and designated combinations, measured in HeLa cells expressing mouse ACE2 (HeLa Mouse-ACE2). Results were calculated from two independent experiments. **(C)** Correlation between IC50 for each antibody and designated combinations against BA.1 pseudovirus measured in HeLa-hACE2 and HeLa Mouse-ACE2. The R^2^ and P values for correlation were 0.9193 and 3.2e-6, determined by two-tailed Spearman correlation. Linear regression of experimental Log IC50 was estimated (solid line) and compared with a hypothetical regression (dotted line) for assumption of equal IC50s in both HeLa-hACE2 and HeLa Mouse-ACE2. Neutralizing activity was significantly higher in HeLa-hACE2 cells than that in HeLa Mouse-ACE2 (P = 0.0001). **(D)** Structural modeling of mouse ACE2 binding to RBD of WT, Delta, Omicron BA.1, and BA.2. Structure of mACE2 binding to a lethal mouse adapted SARS-CoV-2 RBD in grey (PDB:7FDK). Docking of mACE2 onto the Delta RBD in yellow (PDB:7WBQ), or onto Omicron BA.1 and BA.2 RBD in cyan (PDB:7WBP). The major substitutions found in the RBD of studied variants are indicated and those in red showed substantial enhancing effect on entry into HeLa Mouse-ACE2 cell line.

To study the potential impact of Omicron variants on host range and cross-species transmission, we evaluated the ability of ACE2 from five host species to support entry of Delta and Omicron BA.1, BA.1.1, and BA.2 pseudoviruses. HeLa cell lines stably expressing ACE2 molecules from human, mink, mouse, deer, and hamster that have recently been shown susceptible to SARS-CoV-2 infection (44–47) were subjected to analysis. The entry efficiency was measured and presented as fold-changes relative to D614G (**Figure 5A**). Delta acquired substantial ability to infect HeLa cells expressing mink-ACE2 (HeLa Mink-ACE2) and mouse-ACE2 (HeLa Mouse-ACE2), and to a lesser extent deer-ACE2 (HeLa Deer-ACE2). The improved efficiency was about 82.3-fold, 131.7-fold, and 3.4-fold, respectively (**Figure 5A**). Compared to Delta, all three Omicron variants moderately improved their entry efficiency only to infection HeLa Mouse-ACE2, with improve efficiency about 12.4-fold, 8.2-fold, and 8.3-fold, respectively. To pinpoint the potential mutation(s) responsible for enhanced entry efficiency, we generated a total of 21 single-mutant pseudoviruses carrying the specific mutations found within RBD of Delta and the three Omicron variants. Comparing the entry efficiency of these mutant pseudoviruses into HeLa Mouse-ACE2 cells to WT D614G, we found 7 single substitutions (R408S, K417N, Q493R, Q493K, G496S, Q498R, and N501Y highlighted in dark red) substantially improved while 3 (S371L, S371F, and S375F in dark blue) decreased infection (**Figure 5A**). Additional mutations only moderately impacted on the entry efficiency either by improvement (L452R and T478K) or deterioration (T376A, D405N, G446S, and Y505H). This agrees well with the recent reports where either single N501Y, Q493K, or triple K417N-Q493H-N501Y mutations were found in the mouse-adapted SARS-CoV-2 strains, although the triple mutant causes more severe acute respiratory symptoms and mortality in standard laboratory mice (45, 48–50). This is also compatible with the elegant structural analysis on interaction between human ACE2 and RBD of Omicron BA.1 where Q493R substitution was proposed to enhance mouse ACE2 binding through formation of electrostatic interactions with the N31 side chain amide (51). When analysis of entry efficiency into HeLa Mink-ACE2 cells, three substitutions (L452R, T478K, and N501Y) moderately improved whereas two (S371L and S375F) severely compromised the infection (**Figure 5A**). Structurally, these critical mutations are either located on or approximate to the interface between ACE2 and RBD of Delta, Omicron BA.1, and BA.2 (**Figure 5D**). Lastly, substitutions in the inner face (S371L, S371F, S375F, and T376A) and at “mesa” region (G446S and Y505H) of RBD (4) resulted in considerable decrease in entry efficiency to all cell lines studied, suggesting their critical role in upholding overall structure and function for RBD in mediating interaction with ACE2 (**Figures 5A, D**).

To test whether therapeutic antibodies and relevant combinations could inhibit Omicron BA.1 infection of HeLa Mouse-ACE2 cells, serial dilutions of antibodies were incubated with Omicron BA.1 pseudovirus before applied onto HeLa Mouse-ACE2 cells. After 48h, the infected cells were lysed and measured for luciferase-activity. Neutralizing activity was defined as the percent reduction in luciferase activities compared to no antibody controls. As shown in **Figure 5B**, BRII combo was the most potent in inhibiting Omicron BA.1 entry into HeLa Mouse-ACE2 cells, followed by AZ combo, BRII-198, and S309. The rest of antibodies and combinations, however, demonstrated substantial weaker neutralizing activity. Interestingly, linear regression analysis on the IC50 of the tested antibodies and combinations revealed neutralizing activities were more potent in HeLa Human-ACE2 than in HeLa Mouse-ACE2 (P=0.0001), although strong correlation was found between the two systems (**Figure 5C**). This may suggest that Omicron BA.1 spike interacts with mouse ACE2 in a way that is different from that with human ACE2. Nevertheless, given the capacity of BRII combo and AZ combo in inhibiting Omicron BA.1, vaccine capable of inducing antibodies like BRII combo and AZ combo would be expected to provide protection against cross transmission of Omicron BA.1 from human to mouse. Taken together, these results indicate that the Omicron variants acquired mutations in RBD that not only facilitate their escape from antibody neutralization but also potentially expand their host range to mouse and perhaps mink. Active surveillance of these variants in both human and relevant animal species would be required to minimize potential cross-species transmission.

## Discussion

We performed comprehensive analysis on the impact of Omicron BA.1. BA.1.1 and BA.2 on neutralizing activity of therapeutic antibodies and convalescent plasma collected during the initial phase of pandemic in early 2020. Among the VOCs tested, we found Omicron BA.1 and BA.1.1 were the most capable of escaping from neutralization of convalescent plasma from early pandemic and a large number of therapeutic antibodies approved by the regulatory authorities, followed by Beta, BA.2, Gamma, Delta and Alpha. This resistance hierarchy is well correlated with the number of mutations in the NTD and RBD that led to the major antigenic shift in the spike protein. Particularly, Omicron variants has striking number of mutations across the entire spike, including 69-70del and 142-144del in the NTD and triple K417N-E484A-N501Y in RBD that previously found in Beta and Gamma and shown to jeopardize neutralizing activity of most therapeutic antibodies and plasma from convalescent and vaccinated individuals (1–3, 41–43). On top of these, Omicron variants also has additional mutations in the receptor-binding motif (RBM) (N440K, G446S, S477N, T478K, Q493R, G496S, Q498R, Y505H) and on the core domain of RBD (G339D, S371L, S373P, and S375F), which could facilitate Omicron escape from additional antibody and serum neutralization. No complete neutralizing data on BA.3 and BA.4/5 subvariants are currently available. However, given their similar degree of mutations with that found in BA.1, BA.1.1, and BA.2, it is reasonable to speculate that they are also capable of escaping from therapeutic antibodies and plasma of convalescent and vaccinated individuals. Although the specific levels of neutralizing antibody required to confer protection remains uncertain, reductions in antibody titers raises concerns about their protective potentials against all Omicron variants analyzed here.

Among the tested therapeutic antibodies, a few remain active against Omicron variants such as BRII combo, S309, and AZ combo, although the underlying mechanisms might have been different. Despite substantially reduced activity of BRII-196 against Omicron variants, BRII-198 neutralizing and binding activity remained largely unaffected to BA.1, moderately decreased to BA.2, but severely reduced to BA.1.1. However, combination of BRII-196 and BRII-198 managed to maintain neutralizing activity to the Omicron variants up to single-digit μg/mL levels in both in HeLa-hACE2 and Huh7 cell lines. More importantly, BRII combo demonstrated strong protection against Omicron infection in a K18-hACE2 mouse model of SARS-CoV-2 infection, reinforcing its neutralizing activity *in vitro* could be translated into protectivity *in vivo*.

S309 maintained its potency and breadth against Omicron BA.1, BA.1.1 and BA.2, likely attributed to its highly conserved epitope across many VOCs identified so far (1, 2, 4, 43, 51, 52). However, S309 is the only antibody tested here that varied dramatically in its neutralizing activity between two different cell populations. In HeLa-hACE2 cells, S309 generally performed poorly and failed to reach 100% inhibitory effect (IC100) against Omicron variants and 90% inhibitory effect (IC90) against WT. Over expression of ACE2 in the HeLa cells could interfere the very inhibitory mechanism mediated by S309 (39). In this regard, selection of appropriate target cells is therefore fundamental for objectively evaluating antibody neutralization. Unfortunately, the FDA recently announced that the current S309 (sotrovimab) 500 mg dose would not be effective against the Omicron BA.2 subvariant and is therefore no longer authorized to treat COVID-19 in the US against BA.2 infection. In response, GSK/Vir have made public announcement that they are preparing materials and evidence in support of a higher dose of S309 (sotrovimab) for the treatment of Omicron BA.2 subvariant (https://us.gsk.com/en-us/media/press-releases/us-food-and-drug-administration-revises-emergency-use-authorization-for-sotrovimab-due-to-omicron-ba2-subvariant/). The final results have yet been released up till time of writing.

The most unexpected finding is the regaining neutralizing activity of AZ combo against Omicron variants despite each of the individual antibody (COV2-2196 and COV2-2130) markedly reduced or lost neutralizing activity. COV2-2196 and COV2-2130 failed to reach IC90 in HeLa-hACE2 cells so did COV2-2196 in Huh7 cell. However, in both cell types, COV2-2196 and COV2-2130 combo showed impressive synergistic effect and secured the effectiveness of AZ combo against Omicron particularly to subvariant BA.2. The synergistic effect between the two antibodies have also been reported elsewhere (22, 53).

Apart from neutralization escape, Omicron and Delta are found to acquire ability to use mouse ACE2, raising a serious concern of potential transmission to other animal species. Particularly, Omicron demonstrated improved tropism to HeLa Mouse-ACE2, perhaps due to N501Y, Q493K, or triple K417N-Q493H-N501Y mutations previously found to improve replication and cause more severe acute respiratory symptoms and mortality in standard laboratory mice (45, 48–50). The potential mechanism for enhanced entry of Delta remains unclear as K417N, Q493K/R, and N501Y were not found in its RBD (**Supplementary Figure 1**). It needs to be emphasized that our entry studies were conducted on ectopically expressed ACE2 that does not necessarily equal natural infection and transmission in the corresponding animals. However, the same K417N, Q493H/K, and/or N501Y mutations found in mouse adapted SARS-CoV-2 should raise enough concern about the potential spread of these new variants to mice and beyond. Indeed, recently identification of SARS-CoV-2 variants in the mink farm in Denmark (44), free-ranging white-tailed deer (46), and hamsters in pet shops and storage facilities (47) has raised alarming signal about the complexity of host range and cross-species transmission of SARS-CoV-2 variants. Rigorous and thorough monitoring of relevant animals would be required to better understand such complexity and to prevent future transmission to wildlife and spillback to humans.

## Materials and Methods

### Study Approval

The study was approved by the Research Ethics Committee of Shenzhen Third People’s Hospital (2020-084). Entire research was conducted following the rules and regulations of the Chinse government for the protection of human subjects. Blood samples were obtained with informed consent of the study subjects.

### Human Blood Samples

A total of 18 SARS-CoV-2 infected and convalescent patients were enrolled into the study. Their infection status and related demographic information were previously reported (1). All these patients were cured although 7 individuals developed severe pneumonia and the remaining 11 individuals manifested mild symptom. Convalescent blood samples were collected during hospitalization or follow-up visits in Shenzhen Third People’s Hospital, within two months after symptom onset. All blood samples were separated into plasma and peripheral blood mononuclear cells (PBMC) by Ficoll-Hypaque gradient (GE Healthcare) centrifugation. Plasma samples were heat-inactivated at 56 °C for 1h and stored at -80 °C until use.

### Cell Lines

The following cell lines used in the current study were maintained at 37°C in 5% CO2 in Dulbecco’s minimal essential medium (DMEM) containing 10% (v/v) heat-inactivated fetal bovine serum (FBS) and 100 U/mL of penicillin–streptomycin. They included HEK293T cells (ATCC, CRL-3216), Huh7 cells (JCRB, JCRB0403), HeLa cells (ATCC, CCL-2) and HeLa cells expressing ACE2 orthologs kindly provided by Dr. Qiang Ding at School of Medicine Tsinghua University. FreeStyle 293F cells (Thermo Fisher Scientific, R79007) were maintained at 37°C in 5% CO2 in SMM 293-TII expression medium (Sino Biological, M293TII)

### Production of Antibodies

Antibodies approved by the regulatory for clinical use include BRII-196/BRII-198, S309, REGN10933/REGN10987, COV2-2196/COV2-2130 and CB6 were selected for evaluation in the current study. All antibodies except BRII-196/BRII-198 were evaluated using parental IgG antibodies without Fc modification. Apart from BRII-196/BRII-198 derived from our own laboratory, the rest antibodies were synthesized according to the sequences released in Protein Data Bank (PDB) (52, 54–56). Antibodies were produced by co-transfection of the heavy and light chain expression vectors into 293F cells using polyethyleneimine (PEI) (Polysciences). After 96h, antibodies secreted into the cell supernatant were captured by AmMag Protein A Magnetic Beads (Genscript L00695) and eluted by solution buffer Glycine pH 3.0. All antibodies were further purified by gel-filtration chromatography with Superdex 200 High-Performance column (GE Healthcare). The final protein concentrations were determined by nanodrop 2000 Spectrophotometer (Thermo Scientific).

### Production of Pseudoviruses Carrying Wildtype and Mutant Spike Protein

The wildtype pseudovirus used throughout the analysis was the prototype strain (GenBank: MN908947.3) (WT) or with a D614G mutation (D614G). The Alpha variant (Pango lineage B.1.1.7, GISAID: EPI_ISL_601443) included a total of 9 reported mutations in the spike protein (69-70del, 144del, N501Y, A570D, D614G, P681H, T716I, S982A and D1118H). The Beta variant (Pango lineage B.1.351, GISAID: EPI_ISL_700450) included 10 identified mutations in the spike such as L18F, D80A, D215G, 242-244del, S305T, K417N, E484K, N501Y, D614G and A701V. The Gamma variant (Pango lineage P.1, GISAID: EPI_ISL_792681) had 12 reported mutations in the spike including L18F, T20N, P26S, D138Y, R190S, K417T, E484K, N501Y, D614G, H655Y, T1027I and V1176F. The Delta variant (Pango lineage B.1.617.2, GISAID: EPI_ISL_1534938) included 10 reported mutations in the spike such as T19R, G142D, 156-157del, R158G, A222V, L452R, T478K, D614G, P681R, D950N. The Omicron BA.1 variant (Pango lineage BA.1, GISAID: EPI_ISL_6752027) was constructed with 32 mutations in the spike such as A67V, Δ69-70, T95I, G142D/Δ143-145, Δ211/L212I, ins214EPE, G339D, S371L, S373P, S375F, K417N, N440K, G446S, S477N, T478K, E484A, Q493R, G496S, Q498R, N501Y, Y505H, T547K, D614G, H655Y, N679K, P681H, N764K, D796Y, N856K, Q954H, N969K, and L981F. The Omicron BA.1.1 variant (Pango lineage BA.1.1, GISAID: EPI_ISL_7545692) was constructed based on BA.1 variant with additional of R346K substitution. The Omicron BA.2 variant (Pango lineage BA.2, GISAID: EPI_ISL_8515362) was constructed with 29 mutations in the spike such as T19I, 24-26del, A27S, G142D, V213G, G339D, S371F, S373P, S375F, T376A, D405N, R408S, K417N, N440K, S477N, T478K, E484A, Q493R, Q498R, N501Y, Y505H, D614G, H655Y, N679K, P681H, N764K, D796Y, N969K and Q954H.The full-length genes of spike variants were synthesized by Genwiz, Inc. and verified by sequencing. All the mutations in RBD domain of Delta and three Omicron variants were separately introduced into the pcDNA3.1 vector encoding WT D614G SARS-CoV-2. Pseudoviruses were generated by co-transfecting HEK-293T cells (ATCC) with human immunodeficiency virus backbones expressing firefly luciferase (pNL4-3-R-E-luciferase) and pcDNA3.1 vector encoding either wildtype or variant spike proteins. Viral supernatant was collected 48h or 72h later, centrifuged to remove cell lysis, and stored at -80°C until use.

### HeLa Cell Lines Expressing ACE2 From Diverse Host Origin

HeLa cells expressing ACE2 orthologs were kindly provided by Dr. Qiang Ding at Tsinghua University School of Medicine as previously reported (1, 57). The species names and accession numbers of the ACE2 orthologs are listed below: Homo sapiens, NP_001358344.1; Mink, Mustela lutreola, MT560518.1; Mouse, Mus musculus, NP_001123985.1; Chinese hamster, Cricetulus griseus, XP_003503283.1; White-tailed fawn, Odocoileus virginianus texanus, XP_020768965.1. For studying entry efficiency of SARS-CoV-2 variants and WT D614G with single mutation, HeLa-ACE2 cells were added to 96 well plates, mixed with 50 μL of pseudovirus, and analyzed the luciferase activities 48 h after infection using Bright-Glo Luciferase Assay Vector System (Promega Bioscience). Fold changes between the variants and WT D614G were used to estimate the entry efficiency of SARS-CoV-2 variants.

### Antibody and Plasma Neutralization Using Pseudoviruses

Therapeutic antibodies and convalescent plasma were serially diluted before mixing with wildtype or the variants pseudovirus at 37°C for 1h before added onto HeLa-hACE2 cells, Huh7 cells, or HeLa Mouse-ACE2 cells. After 48h, the infected cells were lysed and measured for luciferase-activity. The percent of neutralization was determined by comparing with that of virus control. To ensure properly measuring neutralizing activity, therapeutic antibodies were diluted starting from 100 μg/mL for Omicron pseudovirus and from 10μg/mL for WT pseudovirus. Convalescent plasma was diluted with the highest dilution of 1:60.

### Binding of Antibodies and Convalescent Plasma to Cell Surface-Expressed Wildtype and Omicron Spike Proteins

The entire procedure was conducted as previously published (1, 58). Specifically, HEK 293T cells were transfected with expression plasmids encoding either wildtype or Omicron BA.1, BA.1.1, and BA.2 spike glycoproteins, and incubated at 37°C for 36 h. Cells were digested from the plate with trypsin and distributed onto 96-well plates. Cells were washed twice with 200 µL staining buffer (PBS with 2% heated-inactivated FBS) between each of the following steps. First, cells were stained with each antibody (1 μg/mL), relevant antibody combination (1 μg/mL + 1 μg/mL), diluted convalescent plasma (1:100), or S2-specific monoclonal antibody (1 μg/mL) (MP Biomedicals, Singapore 08720401) at 4°C for 30 min. PE-labeled anti-human IgG Fc (Biolegend 410718), anti-mouse IgG FITC (ThermoFisher Scientific A10673), or anti-his PE secondary antibody (Miltenyi 130120787) was added and incubated at 4°C for 30 min. After extensive washes, the cells were resuspended and analyzed with BD LSRFortassa (BD Biosciences, USA) and FlowJo 10 software (FlowJo, USA). HEK 293T cells with mock transfection were stained as background control. Fold changes in antibody binding were calculated by the ratio between the total fluorescence intensity (TFI) of Omicron over wildtype, normalized by that of S2 specific antibody (nTFI). TFI was calculated by multiplying the mean fluorescence intensity (MFI) and the number of positive cells in the selected gates.

### Antibody Protection in hACE2 Transgenic Mice

Animal experiments were conducted in a Biosafety Level 3 (BSL-3) facility in accordance with the National University of Singapore (NUS) Institutional Animal Care and Use Committee (IACUC) (protocol no. R20-0504), and the NUS Institutional Biosafety Committee (IBC) and NUS Medicine BSL-3 Biosafety Committee (BBC) approved SOPs. Eight-week-old female K18-hACE2 transgenic mice (InVivos Ptd Ltd, Lim Chu Kang, Singapore) were used for this study. The mice were housed and acclimatized in an ABSL-3 facility for 72 h prior to the start of the experiment. K18-hACE2 transgenic mice were subjected to BRII combo (10 + 10 mg/kg) delivered through intraperitoneal injection a day prior to infection (n=12) or left untreated (n=12). The BRII combo was validated in live virus neutralization assay with IC50 0.168 μg/ml and IC90 0.828 μg/ml. The viral challenge was conducted through intranasal delivery in 25 μl of 1.7×10^3^ PFU of the infectious SARS-CoV-2 Omicron BA.1. Body weights were measured prior to infection as baseline and monitored daily throughout the following 14 days. Mice were euthanized when their body weight fell below 75% of their baseline body weight. Six mice from each experimental group were sacrificed 3 days post inoculation, with lung and brain tissues harvested. Each organ was halved for the plaque assay and histology analysis, respectively.

For virus titer determination, supernatants from homogenized tissues were diluted 10-fold serially in DMEM supplemented with antibiotic and antimycotic and added to Vero E6 cells in 12-well plates. The inoculum was removed after 1 h of incubation for virus adsorption. Cells were washed once with PBS before 1.2% MCC-DMEM overlay media supplemented with antibiotic and antimycotic was added to each well. Then cells were incubated at 37°C, 5% CO2 for 72 h for plaque formation. The cells were fixed in 10% formalin overnight and counterstained with crystal violet. The number of plaques was determined, and the virus titers of individual samples were expressed in logarithm of PFU per organ.

For histopathological analyses, lung lobes were fixed in 3.7% formaldehyde solution prior to removal from BSL-3 containment. The tissues were routinely processed, embedded in paraffin blocks (Leica Surgipath Paraplast), sectioned at 4-μm thickness, and stained with H&E (Thermo Scientific) following standard histological procedures. For immunohistochemistry, sections were deparaffinized and rehydrated, followed by heat-mediated antigen retrieval, quenching of endogenous peroxidases and protein blocking. Sections were then covered with rabbit anti-SARS-CoV-2 N protein monoclonal antibody (Abcam; 1:1000) for 1 h at room temperature. Subsequently, sections were incubated with rabbit-specific HRP polymer (secondary antibody), visualized using chromogenic substrate DAB solution (Abcam), and counterstained with hematoxylin.

### Structural Modeling of Mouse ACE2 Binding to RBD

The complex structure of mACE2 bound to a mouse adapted SARS-CoV-2 RBD (PDB:7FDK) was used to indicate critical residues that affected interaction between mACE2 and Delta RBD (PDB:7WBQ), and Omicron BA.1 and BA.2 RBD (PDB:7WBP). Pymol software was utilized for construction and demonstration of structural models.

### Statistical Analysis

The technical and independent experiment replicates were indicated in the figure legends. Half-maximal inhibitory concentration (IC50) of mAb or dilutions (ID50) of convalescent plasma were calculated by the equation of four-parameter dose inhibition response using Graphpad Prism 8.0. The fold change of the variants relative to wildtype in neutralization were calculated by simple division of respective IC50 or ID50 values. The overall fold change in plasma neutralization to mutant over D614G pseudovirus was calculated by the geometric mean of the ID50 value of the 18 plasma samples. The significance of neutralizing and binding activities of convalescent plasma against each mutant pseudovirus relative to D614G was estimated using the paired t test by graphpad 8.0. Log IC50 of antibodies and the designated combinations between different cell types were fitted into linear regression model, and Spearman correlation was calculated by graphpad 8.0. The statistical differences between fitted regression model with hypothetical regression model was calculated using F test.

## Data Availability Statement

The original contributions presented in the study are included in the article/**Supplementary Material**. Further inquiries can be directed to the corresponding authors.

## Author Contributions

LZ and JC conceived and designed the study. RW, QZ, RZ, and ZA performed most of the experiments with assistance from PC, YW, JH, and XS. BJ provided plasmids of some of antibody production. QD provided HeLa cell lines expressing ACE2 from diverse origin. ZZ provided clinical care and management of infected patients, and particularly the recruitment and following up the study subjects. RW, QZ, RZ, and LZ had full access to data in the study, generated figures and tables, and take responsibility for the integrity and accuracy of the data presentation. LZ and JC wrote the manuscript. All authors contributed to the article and approved the submitted version.

## Funding

This study was funded by the National Key Plan for Scientific Research and Development of China (2020YFC0848800, 2020YFC0849900, 2021YFC0864500 and 2020YFC0861200), the National Natural Science Foundation (92169205, 81530065, 81661128042, 9216920007 and 32000661), Beijing Municipal Science and Technology Commission (D171100000517001, D171100000517003 and Z201100005420019), the Science and Technology Innovation Committee of Shenzhen Municipality (202002073000002 and JSGG20200807171401008), Beijing Advanced Innovation Center for Structural Biology, Tsinghua University Scientific Research Program (20201080053 and 2020Z99CFG004), Tencent Foundation, Shuidi Foundation, TH Capital, and the National Science Fund for Distinguished Young Scholars (82025022), Singapore National Medical Research Council Centre Grant Program (CGAug16M009), NUHSRO/2020/066/NUSMedCovid/01/BSL3 Covid Research Work, NUHSRO/2020/050/RO5+5/NUHS-COVID/4, Ministry of Education, Singapore MOE2017-T2-2-014; Singapore NMRC Centre Grant Program – Diabetes, Tuberculosis and Neuroscience CGAug16M009, Singapore Ministry of Health MOH-COVID19RF2-0001. The funders had no role in study design, data collection, data analysis, data interpretation or writing of the report.

## Conflict of Interest

The authors have filed patent applications on antibodies BRII-196 and BRII-198 described in the manuscript. LZ, QZ, and XS are shareholders of TSB Therapeutics.

## Publisher’s Note

All claims expressed in this article are solely those of the authors and do not necessarily represent those of their affiliated organizations, or those of the publisher, the editors and the reviewers. Any product that may be evaluated in this article, or claim that may be made by its manufacturer, is not guaranteed or endorsed by the publisher.
