# Supplementary material for: SARS-CoV-2 Omicron Variants Reduce Antibody Neutralization and Acquire Usage of Mouse ACE2
Source: Front Immunol. 2022 Jun 17;13:854952. doi: 10.3389/fimmu.2022.854952 (PMC9247160; doi:10.3389/fimmu.2022.854952)
Supplement: Supplementary file 1 [file DataSheet_1.docx]

Supplementary Materials for

**SARS-CoV-2 Omicron variants reduce antibody neutralization and acquire usage of mouse ACE2**

Ruoke Wang^1,2†^, Qi Zhang^1†^, Rui Zhang^1†^, Zhen Qin Aw^3,4,5†^ Peng Chen^1†^, Yi Hao Wong^3,4,5^, Junxian Hong^1^, Bin Ju^6,7^, Xuanling Shi^1^, Qiang Ding^8,9^, Zheng Zhang^6,7^, Justin Jang Hann Chu^3,4,5^*, and Linqi Zhang^1,10,11^*

Correspondence to: miccjh@nus.edu.sg, [zhanglinqi@tsinghua.edu.cn](mailto:zhanglinqi@tsinghua.edu.cn)

**This file includes:**

Supplementary Figure 1-4


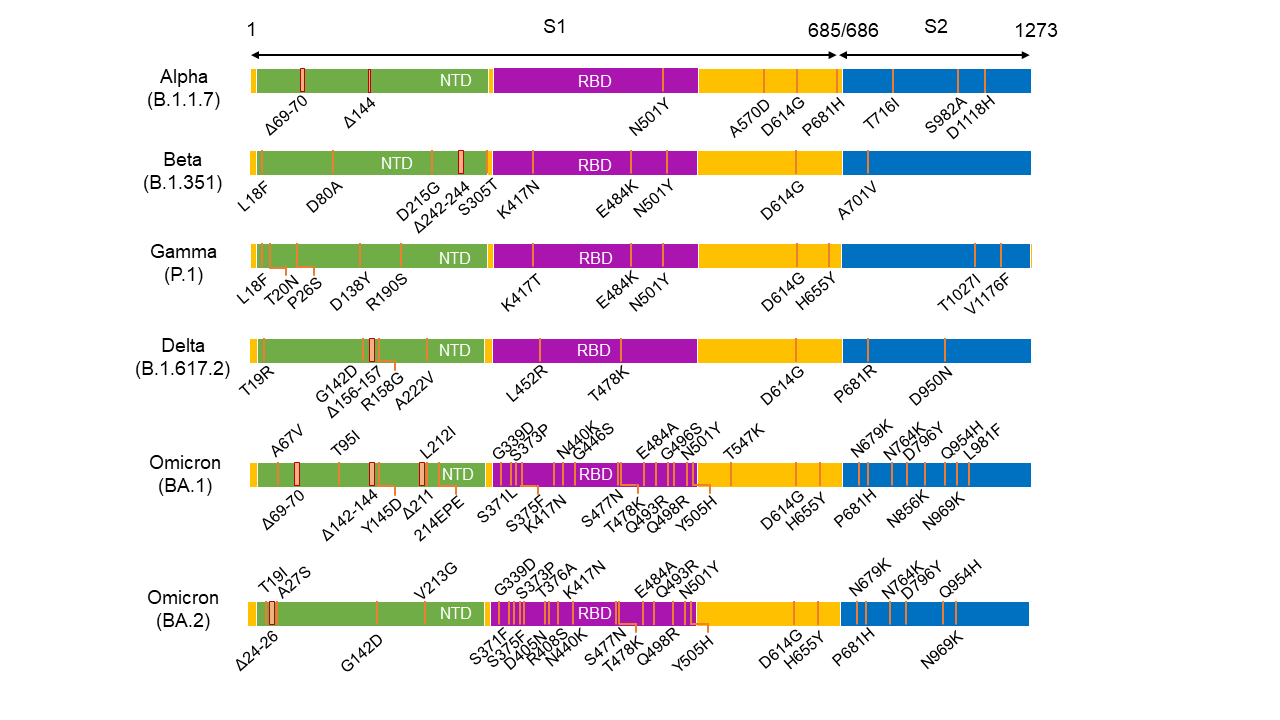


**Supplementary Figure 1, related to Figure 1.** Amino acid substitutions in the spike of SARS-CoV-2 variants of concern. The name and corresponding Pango lineage for each variant are indicated on the far left, together with specific residue substitutions along the full-length spike protein. The N-terminal domain (NTD) and receptor-binding domain (RBD) in the S1 region as well as S2 region are indicated, along with the residue numbers spanning the S1 and S2 regions. The yellow vertical lines indicate single residue substitutions and insertions whereas red boxes indicate the deletions.

**Supplementary Figure 2, related to Figure 1 and 3. Neutralization of SARS-CoV-2 BA.1 live virus by BRII combo, BRII-196 and BRII-198.** BA.1 live virus was tested against serial dilutions of BRII combo, BRII-196 and BRII-198. Serial dilutions of the test antibodies were conducted, mixed with 50 PFU infectious SARS-CoV-2 in 96-well plates, and incubated for 1 h at 37°C. The mixtures were then transferred into 12-well plates, seeded with A549-hACE2 cells, and allowed absorption for 1 h at 37°C. The inocula were then removed and the wells washed once with PBS before adding the overlay media [1 ml DMEM containing 1.2% microcrystalline cellulose (MCC)]. The plates were then incubated at 37°C, 5% CO_2_ for 72 h for plaque formation. Cells were fixed in 10% formalin overnight before counterstaining with crystal violet. The virus titer of each dilution was determined through the number of plaques formed and expressed in neutralization percentage in comparison to no antibody control.


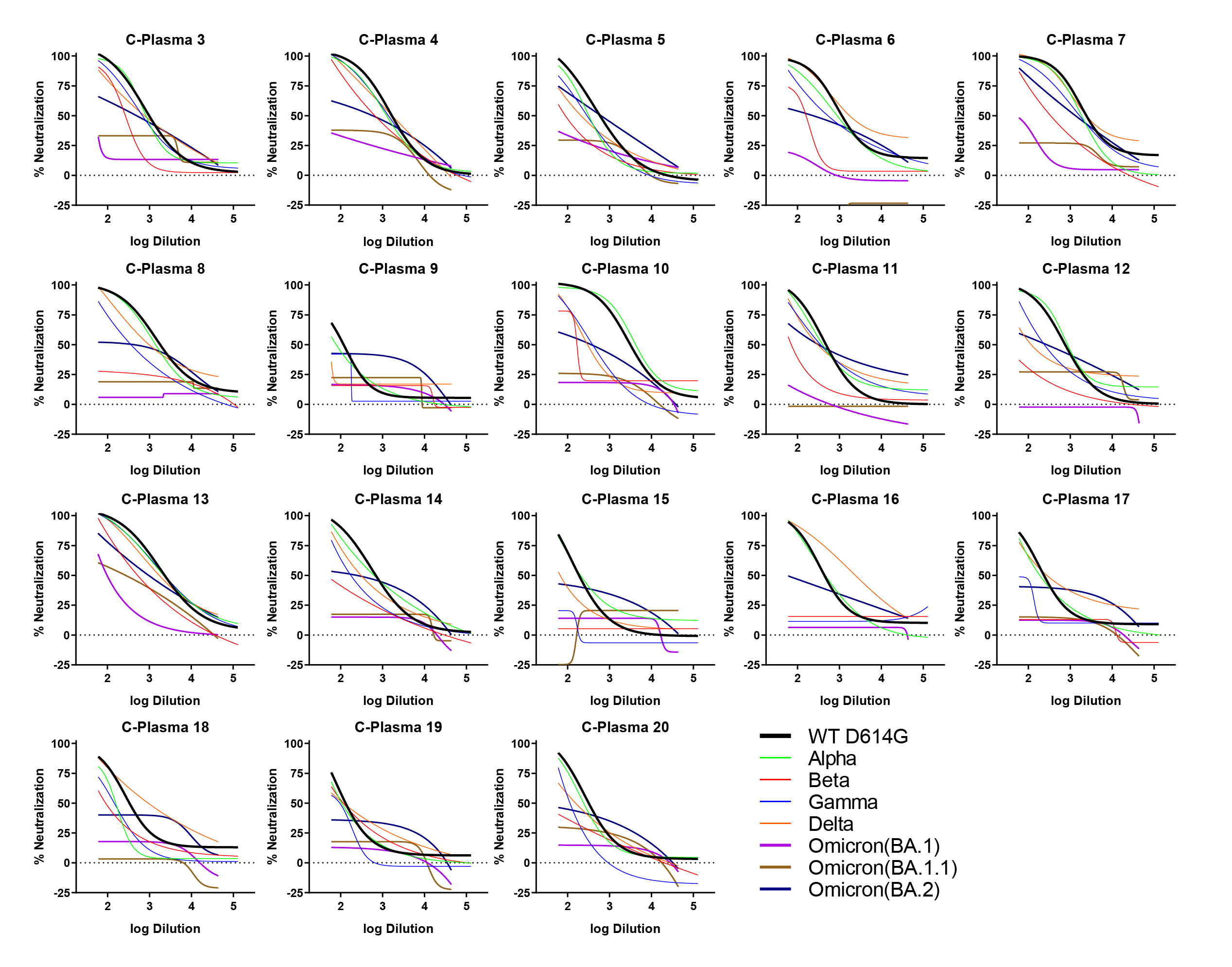


**Supplementary Figure 3, related to Figure 4. Neutralization of SARS-CoV-2 VOCs by each convalescent plasma.** Pseudoviruses bearing the Alpha, Beta, Gamma, Delta, and Omicron spike were tested against serial dilutions of convalescent plasma. Neutralization activity was defined as the percent reduction in luciferase activity relative to no serum controls. The ID50 and fold changes between each variant and D614G pseudovirus were calculated to estimate the resistance levels shown in Figure 4. Results were calculated from at least two independent experiments.


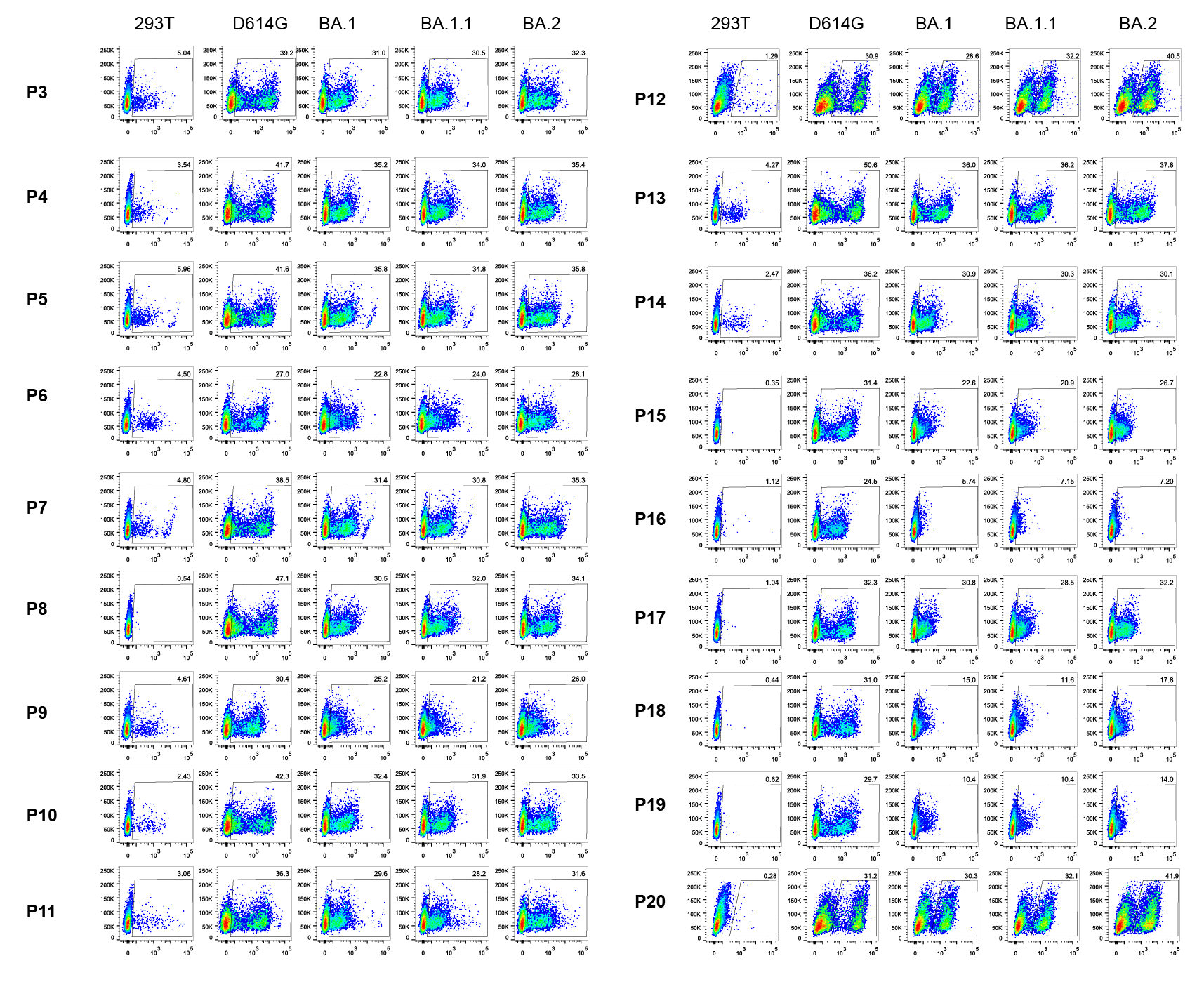


**Supplementary Figure. 4, related to Figure 4.** **Plasma binding to spikes of Omicron BA.1, BA.1.1, and BA.2 expressed on the cell surface.** Binding of plasma samples to WT D614G, Omicron BA.1, BA.1.1, and BA.2 spike proteins expressed on the surface of HEK293T, measured by flow cytometry. The numbers highlighted in the gates represent the percent of positive cells detected by indicated plasma samples. The result shown was representative of three independent experiments.
